# Purine nucleosides: endogenous neuroprotectants in hypoxic brain

**DOI:** 10.1111/j.1471-4159.2012.07692.x

**Published:** 2012-05

**Authors:** Bettina Thauerer, Stephanie zur Nedden, Gabriele Baier-Bitterlich

**Affiliations:** Division of Neurobiochemistry, Biocenter Department, Medical University of InnsbruckInnsbruck, Austria

**Keywords:** adenosine, guanosine, hypoxia, inosine, neuron, purine nucleosides

## Abstract

**Abstract:**

Even a short blockade of oxygen flow in brain may lead to the inhibition of oxidative phosphorylation and depletion of cellular ATP, which results in profound deficiencies in cellular function. Following ischemia, dying, injured, and hypoxic cells release soluble purine-nucleotide and -nucleoside pools. Growing evidence suggests that purine nucleosides might act as trophic factors in the CNS and PNS. In addition to equilibrative nucleoside transporters (ENTs) regulating purine nucleoside concentrations intra- and extracellularly, specific extracellular receptor subtypes for these compounds are expressed on neurons, glia, and endothelial cells, mediating stunningly diverse effects. Such effects range from induction of cell differentiation, apoptosis, mitogenesis, and morphogenetic changes, to stimulation of synthesis and/or release of cytokines and neurotrophic factors under both physiological and pathological conditions. Multiple signaling pathways regulate the critical balance between cell death and survival in hypoxia–ischemia. A convergent pathway for the regulation of multiple modalities involved in O_2_ sensing is the mitogen activated protein kinase (p42/44 MAPK) or (ERK1/2 extracellular signal-regulated kinases) pathway terminating in a variety of transcription factors, for example, hypoxia-inducible factor 1α. In this review, the coherence of purine nucleoside-related pathways and MAPK activation in the endogenous neuroprotective regulation of the nervous system's development and neuroplasticity under hypoxic stress will be discussed.

## Hypoxia in brain

**Figure 1:**
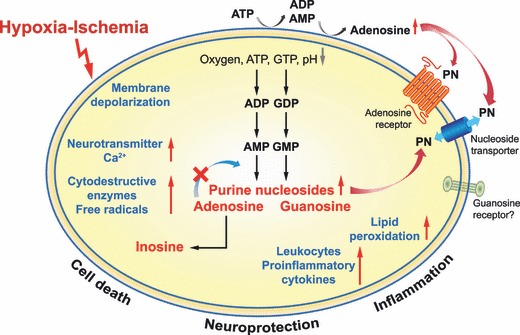
Biochemistry of ischemia–reperfusion injury. Hypoxic–ischemic brain injury starts with the insult but extends into a recovery-reperfusion period (Barone and Feuerstein 1999; Lipton 1999; White *et al.* 2000; Hertz 2008; Macrez *et al.* 2011). In case of prolonged ischemia, restricted blood flow leads to a reduction in ATP, causing severe impairment of cellular function by disruption of ATP-dependent processes. A key incidence is the increase in intracellular calcium, which is responsible for the release of neurotransmitters such as glutamate and the activation of many cytocidal enzymes. Activated endonucleases then lead to DNA damage and apoptosis. Though restoration of seized blood flow and oxygen delivery is essential for organ survival, damage is potentially amplified during this period by oxygen sensitive mechanisms, for example, by the activity of pro-inflammatory cytokines (Barone and Feuerstein 1999; Lipton 1999; White *et al.* 2000; Hertz 2008; Macrez *et al.* 2011). In parallel, hypoxia leads to the decreased production and enhanced breakdown of purine nucleotides to purine nucleosides (PN) (Jurkowitz *et al.* 1998; Sitkovsky *et al.* 2004; Fredholm *et al.* 2007; Fredholm 2010), which may enter/leave cells via bidirectional nucleoside transporters (ENTs) or in the case of adenosine and inosine directly bind to adenosine receptors (Fredholm *et al.* 1994, 2001a; Schulte and Fredholm 2003b). To date it is not clear, whether the protective effect of guanosine is at least partly arbitrated by adenosine or adenosine receptors (Ciccarelli *et al.* 2000; D'Alimonte *et al.* 2007), or is mediated by its own specific G-coupled receptors (Traversa *et al.* 2003; Rathbone *et al.* 2008).

In acute neurological conditions such as stroke severe injuries to the CNS occur (Honig and Rosenberg 2000) and stroke is the second most common cause of death and a major cause of long-term disability worldwide (Macrez *et al.* 2011). Hippocampus and cerebellar cortex are particularly sensitive to ischemia (Yue *et al.* 1997). Hypoxic–ischemic insult generally causes necrosis, although in most cases there exists also a process of delayed and apoptotic type injury in the region (penumbra) surrounding the area of most severe damage (Honig and Rosenberg 2000; Yuan and Yankner 2000; Schaller *et al.* 2003; Lo 2008). Lately, it was considered that this degeneration might be better regarded as an 'apoptosis-necrosis cell death continuum' (Northington *et al.* 2011). Neurons in the adult mammalian CNS, which are injured by stroke normally fail or have only limited ability to regenerate axons, which causes long lasting disabilities in sensory, motor, or cognitive functions (Benowitz and Carmichael 2010). In addition, cell death in the brain leads to the subsequent release of endogenous molecules termed 'damage-associated molecular patterns' from dying cells, triggering further cascades of inflammatory events that both have deleterious but also beneficial effects (Sitkovsky *et al.* 2004; Chen and Nunez 2010). As an immediate result, a disrupted microcirculation leads to local tissue hypoxia associated with an impaired adenosine 5′-triphosphate (ATP) production and energy status of neurons and glia. This is the basis of further insults including increased calcium, release of glutamate, synthesis of enzymes involved in free radical production and the accumulation of leukocytes (Barone and Feuerstein 1999; Lipton 1999; White *et al.* 2000; Hertz 2008) (Fig. 1). In the hope to improve clinical outcome after stroke, remarkable progress in understanding its pathophysiology has been made in the past 10 years and basic research yielded numerous pharmacologic agents leading to the identification of more than 1000 molecules with brain-protective effects from experimental models and to the implementation of more than 250 clinical trials. However, none has so far successfully completed phase III clinical development and the only acute pharmacological treatment approved to date is tissue plasminogen activator and aspirin, other antiplatelets, and anticoagulants are used as preventative therapy (Young *et al.* 2007; Ginsberg 2009; Moskowitz *et al.* 2010; Albers *et al.* 2011; Macrez *et al.* 2011).

## Purine nucleosides in hypoxia

Following hypoxia–ischemia, dying, injured, and hypoxic cells release soluble purine- nucleotide and -nucleoside pools (Ciccarelli *et al.* 1999; Dale *et al.* 2000; Latini and Pedata 2001), normally regulated by ENTs, ectonucleotidases and ecto-adenosine deaminase (Delaney *et al.* 1998; Zimmermann *et al.* 1998; Dunwiddie and Masino 2001; Frenguelli *et al.* 2003; Fredholm *et al.* 2011; Ipata *et al.* 2011; Zhang *et al.* 2011). Purine nucleoside-mediated effects in hypoxia are therefore exceptionally interesting due to their endogenous regulatory mechanisms in stress situations. Growing evidence suggests that purine nucleosides, which may remain elevated for days after the insult (Uemura *et al.* 1991), might also act as trophic factors in both the CNS and PNS (Neary *et al.* 1996; Rathbone *et al.* 1999). In addition to ENTs regulating purine nucleoside concentrations, specific extracellular receptor subtypes for these compounds are expressed on neurons, glia, and endothelial cells, where they mediate strikingly different effects. Such effects range from induction of cell differentiation, apoptosis, mitogenesis, and morphogenetic changes, to stimulation of synthesis and/or release of cytokines and neurotrophic factors under both physiological and pathological conditions (Fields and Burnstock 2006; Burnstock 2008). Nucleosides, for example, adenosine, inosine and guanosine are therefore likely to be involved in the regulation of the nervous system's development and plasticity (Neary *et al.* 1996).

Adenosine is formed by stepwise dephosphorylation of ATP (Zimmermann 2000) and to a minor extent from hydrolysis of *S*-adenosyl homocysteine (Deussen *et al.* 1989). Normally, it is present in body fluids in concentrations 20–200 nM but in response to stress, for example, hypoxia, ischemia, inflammation and trauma, elevated levels of adenosine, up to 300 μM are produced and released (Fredholm *et al.* 2001a; Fredholm 2007, 2010; Burnstock 2008; Lopes *et al.* 2011). This observed increase in extracellular adenosine is due to a decreased production of intracellular ATP, accumulation of AMP, enhanced dephosphorylation of adenine nucleotides to adenosine by cytosolic-5′-nucleotidase and inhibition of adenosine kinase (Sitkovsky *et al.* 2004; Sitkovsky 2009) and liberation from cells via nucleoside transporters (Pastor-Anglada *et al.* 2001). However, extracellular adenosine accumulates through the activities of local tissue hypoxia-up-regulated ectonucleotidase activity (Braun *et al.* 1998) (Fig. 1). In conditions of profound hypoxia these could depict significant sources of extracellular adenosine, which was shown in other cell systems to generate immunosuppressive loops (Ohta and Sitkovsky 2001; Sitkovsky *et al.* 2004; Deaglio *et al.* 2007; Fredholm 2007; Sitkovsky 2009).

Adenosine then acts as a powerful endogenous neuroprotectant and intra- and intercellular messenger during ischemia-induced energy failure (Dunwiddie and Masino 2001; Fredholm *et al.* 2005b) by decreasing neuronal metabolism and increasing cerebral blood flow and there is reduction in the release of excitotoxic neurotransmitters, attenuation of NMDA receptors, vasorelaxation, and anti-inflammatory effects (Sciotti *et al.* 1992; Yawo and Chuhma 1993; Soricelli *et al.* 1995; Johansson *et al.* 2001; Ohta and Sitkovsky 2001; Li *et al.* 2006; Kusano *et al.* 2010). But most importantly, there is, at least in animal models, an impressive reduction of neuronal damage and mortality (von Lubitz 1999). Adenosine also showed neuroprotective effects in *in vitro* models of hypoxic neuronal cells (Bocklinger *et al.* 2004; Heftberger *et al.* 2005; Tomaselli *et al.* 2005a; b; Tomaselli *et al.* 2008; zur Nedden *et al.* 2008).

Adenosine effects are mediated by specific receptors (Rudolphi *et al.* 1992; Sweeney 1997; Kobayashi *et al.* 1998; von Lubitz 1999; Fredholm *et al.* 2001a). In brain, high membrane adenosine receptor expression levels are found (Fredholm *et al.* 2005b; Hasko *et al.* 2005; Wei *et al.* 2010), and stimulation of adenosine receptors was hypothesized to result in an effective treatment of stroke (Dunwiddie and Masino 2001; Laubach *et al.* 2011). As discussed before, extracellular adenosine accumulates in inflamed areas with damaged microcirculation, diminished blood supply, and low oxygen tension. Under such conditions, adenosine serves as a marker of collateral immune damage and supports the prevention of additional injury through inhibition of activated immune cells (Sitkovsky *et al.* 2004; Sitkovsky 2009). Adenosine deaminase (converting adenosine to inosine), adenosine kinase (phosphorylates adenosine to 5′-AMP) and nucleoside transporters, are responsible for an extremely short half-life of adenosine in circulation (Fredholm *et al.* 2001a, 2011; Eltzschig 2009) and therefore some of its effects, are apparently due to its metabolites as was reported, for example, for inosine (Haun *et al.* 1996).

Inosine is formed by deamination of adenosine, mainly at high intracellular concentrations, which are associated with hypoxia, ischemia and other forms of cellular stress (Hasko *et al.* 2004). Inosine may be formed intra- and extracellularly and shunted across the cell membrane via ENTs (Pastor-Anglada *et al.* 2001) (Fig. 1). Inosine concentrations up to 6 μM have been detected in human myocardial ischemia, and many times higher concentrations may be observed in experimental models of ischemia–reperfusion injury (Hasko *et al.* 2004). Initially, inosine did not attract the same interest as adenosine. Yet, inosine was shown to effect neuronal (Benowitz *et al.* 1998; Litsky *et al.* 1999; Bocklinger *et al.* 2004; Heftberger *et al.* 2005; Tomaselli *et al.* 2005a, b, 2008; zur Nedden *et al.* 2008) and glial (Haun *et al.* 1996; Jurkowitz *et al.* 1998) cell viability and neurite outgrowth in cells subjected to glucose deprivation and/or mitochondrial respiratory chain inhibition or challenged with low oxygen. Moreover, inosine was shown to stimulate neurons to extend new projections to denervated areas in adult rats with unilateral cortical infarcts (Chen *et al.* 2002). Inosine was also shown to exert multiple anti-inflammatory effects such as reduction of the production of pro-inflammatory cytokines such as tumor necrosis factor-alpha, macrophage inflammatory protein-2 and Il-6 (Hasko *et al.* 2004). These findings, coupled with the fact that inosine has very low toxicity, suggested that this agent may be useful in the treatment of inflammatory/ischemic diseases, and might help to restore essential circuitry after injury to the CNS (Jurkowitz *et al.* 1998; Benowitz *et al.* 1999, 2002; Chen *et al.* 2002; Hasko *et al.* 2004; Zai *et al.* 2009; Benowitz and Carmichael 2010).

Guanosine is metabolized from guanosine 5′-triphosphate (GTP) and guanosine 5′-monophosphate (GMP) (Schmidt *et al.* 2007) and is present in the brain under both physiological and pathological conditions (Uemura *et al.* 1991). In analogy to ATP, GTP concentrations decrease in ischemic tissue (Kinouchi *et al.* 1990) and guanosine concentrations showed significant increases at 2 h to 7 days (Uemura *et al.* 1991). Guanine derivatives may reach threefold higher levels than adenine-derivatives in cell injuries like hypoxia and hypoglycemia (Ciccarelli *et al.* 1999). Guanine-based purines are released from neurons and astrocytes (Rathbone *et al.* 2008). As discussed (Rathbone *et al.* 2008), extracellular guanosine stimulates mitosis, synthesis of trophic factors, and cell differentiation, including neuritogenesis, is neuro- and glia-protective, and reduces apoptosis (Gysbers and Rathbone 1996a, b; Benowitz *et al.* 1998; Jurkowitz *et al.* 1998; Rathbone *et al.* 1998, 2008, 2011; Litsky *et al.* 1999; Ciccarelli *et al.* 2000; Frizzo *et al.* 2002; Bau *et al.* 2005; Tomaselli *et al.* 2005b; Ballerini *et al.* 2006; Jiang *et al.* 2007; Schmidt *et al.* 2007; Chang *et al.* 2008; Oleskovicz *et al.* 2008; Su *et al.* 2010; Thauerer *et al.* 2010; Dal-Cim *et al.* 2011).

## Purine nucleoside receptors

Adenosine receptors (AR) belong to the superfamily of G-protein-coupled receptors characterized by seven transmembrane helices (Palmer and Stiles 1995). There are four G-protein-coupled ARs, namely A1R, A2AR, A2BR and A3R, all of them expressed in brain, with A1R and A2AR being the physiologically more important subtypes (Fredholm *et al.* 2001a, 2005a; Abbracchio *et al.* 2009; Wei *et al.* 2010). ARs, via its alpha subunit, either stimulate (*G*_s_) or inhibit (*G*_i_) adenylate cyclase, the enzyme that catalyzes the formation of cAMP, whereby A1R and A3R interact with G_i_/ G_o_ proteins, and A2A and A2B with G_s_ (van Calker *et al.* 1979; Zhou *et al.* 1992; Palmer and Stiles 1997; Fredholm *et al.* 2001a). In addition to the classical adenylate cyclase–cAMP-protein kinase A signaling pathway, it is now apparent that other pathways, such as phospholipase C, Ca^2+^- and mitogen-activated protein kinases (MAPKs), are also relevant (Linden 1991; Abbracchio *et al.* 1995; Fredholm *et al.* 2001a; Schulte and Fredholm 2003b; Tomaselli *et al.* 2008).

The A1 high affinity receptor albeit expressed throughout the body reaches highest levels in brain especially in neurons of cortex, hippocampus, cerebellum and dorsal horn of spinal cord, eye, adrenal gland, and atria (Fredholm *et al.* 2005b; Wei *et al.* 2010) at pre-synaptic and post-synaptic sites (Rebola *et al.* 2003). A1R stimulation generally suppresses neuronal activity and efficiently controls the release of all the classical neurotransmitters (glutamate, acetylcholine and serotonin), leading to the idea that A1Rs mainly fulfill a synaptic neuromodulatory role, particularly in excitatory nerve terminals in the brain (Dunwiddie and Masino 2001; Cunha 2005; Fredholm *et al.* 2005a; b; Wei *et al.* 2010). The expression of the high affinity A2AR is highest in brain in dopamine-rich regions, the striato-pallidal GABAergic neurons and olfactory bulb (Peterfreund *et al.* 1996; Wei *et al.* 2010). Evidence indicates that activation of A2AR exerts damaging as well as protective effects in brain ischemia (Dai and Zhou 2011). The preferred partner of A2AR-mediated activation is *G*_s_, except in striatum, where A2AR interacts with *G*_olf_, whereby both result in coupling to its canonical protein kinase A (PKA)-activating pathway (Fredholm *et al.* 2007). Stimulation of the A2AR activates the Ras/RAF-1/MEK/MAPK signaling through PKA-dependent and PKA-independent pathways via Src-and Sos-mediated mechanisms (Schulte and Fredholm 2003b). Hypoxic conditions were shown to up-regulate A1R and A2AR (Kobayashi and Millhorn 1999; Lai *et al.* 2005; Podhraski *et al.* 2005). Selective AR agonists and antagonists were created and extensively reviewed recently (Lopes *et al.* 2011; Muller and Jacobson 2011).

Adenosine is a full agonist at all these receptors, whereas inosine can act as a partial agonist in functional assays at A1R and A3R (Jin *et al.* 1997; Fredholm *et al.* 2001a; Hasko *et al.* 2004) and initiate intracellular signaling events (Jin *et al.* 1997; Hasko *et al.* 2000, 2004; Fredholm *et al.* 2001b). Recent findings showed inosine-mediated stimulatory effects in the predominantly A2AR-positive neuronal PC12 cell line (zur Nedden *et al.* 2008; Tomaselli *et al.* 2008). However, it remains to be seen whether these systemic immunomodulatory effects are the consequences of direct binding of inosine to A2AR. Even more controversial is the question, whether the protective effect of guanosine is at least partly arbitrated by adenosine or its receptors (Ciccarelli *et al.* 2000; D'Alimonte *et al.* 2007), or is mediated by its own specific G-coupled receptors (Traversa *et al.* 2003; Rathbone *et al.* 2008). Recently, results again suggest that guanosine, 6-thioguanosine, and their derivatives activate a G-protein-coupled receptor that is different from the well-characterized AR (Volpini *et al.* 2011). Our own data suggest at least a supporting role for the A2AR in guanosine-mediated signal transduction in neurite formation ((Thauerer *et al.* 2010) and B. Thauerer, unpublished data).

## Nucleoside transporters

During metabolic stress like hypoxia, intracellular adenosine is formed at the expense of ATP, leaves cells via nucleoside transporters and activate ARs (Fredholm *et al.* 2005a). Bidirectional transporters allow purine nucleosides to gain access to the intracellular space (Pastor-Anglada *et al.* 2001; Parkinson *et al.* 2005, 2011; King *et al.* 2006; Takahashi *et al.* 2010; Sebastiao 2011). Alternatively, ATP may be released from cells by cell lysis, exocytosis, transporters or channels, and dephosphorylated extracellularly to adenosine (Neary 2005). The increase in inosine during hypoxia was reported to be largely due to, either extracellular degradation of adenosine (Frenguelli *et al.* 2003), or else to the intracellular formation of inosine and subsequent release by ENTs (Parkinson and Xiong 2004). Likewise guanosine was shown to be transported into neurons and astrocytes by nucleoside transporters (Nagasawa *et al.* 2007).

## Purine nucleoside-mediated neuroprotection and neuroregeneration

Evaluation of the effects of AR agonists and antagonists in stroke models indicates that adenosine acting through A1R has neuroprotective effects (Rudolphi *et al.* 1992; Sweeney 1997; von Lubitz 1999), probably by control of glutamate release and inhibiting excitatory synaptic neurotransmission in the brain during hypoxia (Wei *et al.* 2010). In contrast, activation of A2AR may enhance neuronal damage, as mice lacking these receptors exhibited reduced damage following focal ischemia (Chen *et al.* 1999). Results by others (Kobayashi *et al.* 1998), however indicated that hypoxia-induced membrane responses of PC12 cells are likely to be mediated via activation of the A2AR.

Administration of adenosine to the brain at times of stroke was shown to ameliorate damage (Kitagawa *et al.* 2002), and transgenic over-expression of adenosine kinase, leads to increased vulnerability to ischemia-induced cell death (Pignataro *et al.* 2007). It is now at large believed and confirmed by genetic knockout models, that elevated extracellular adenosine levels exert an overall neuroprotective effect in injured brain; however, because of complex organ- and injury-type specific responses precise predictions are still difficult (Wei *et al.* 2010). Correspondingly, stroke animals receiving inosine pre-treatment demonstrated a higher level of locomotor activity and less cerebral infarction (Shen *et al.* 2005). Also guanosine was shown to prolong rat survival and decrease both neurological deficits and tissue damage resulting from middle cerebral artery occlusion (MCAo) (Chang *et al.* 2008). Other strategies to improve outcome after stroke-induced injuries are considering a potential-reinnervation of brain regions that are devoid of their normal inputs. Along this line, adenosine and guanosine were shown to inhibit injury-induced axonal degeneration in cultured dorsal root ganglion (DRG) neurons (Press and Milbrandt 2009). Also inosine, proved to alter gene expression in neurons and enhance the ability of neurons to form new connections on the side of the spinal cord that lost its normal innervation, and to restore skilled behavior formerly mediated by the damaged area, thus revealing their potential to modulate circuit remodeling that might recover lost functions (Zai *et al.* 2009). Together these findings give rise to the hope of new therapeutical approaches for the improvement of hypoxia/ischemia-induced plasticity and to lessen neuronal damage in stroke (Zai *et al.* 2009; Benowitz and Carmichael 2010).

## Hypoxia signaling

Oxygen sensing and adaptation is achieved by a variety of molecules whose effects are complexly interwoven, and sophisticated mechanisms have evolved that regulates gene expression and the critical balance between cell death and survival during hypoxia/ischemia (Seta *et al.* 2002). Those include the Ca^2+^-calmodulin pathway, the 3′–5′ adenosine monophosphate (cAMP)-PKA pathway, the MAPK pathway, the stress-activated protein kinase (also known as p38 kinase) pathway, and the phosphatidylinositol 3-kinase-Akt pathway (Conrad *et al.* 2001; Bickler and Donohoe 2002; Seta *et al.* 2002; Seta and Millhorn 2004; Semenza 2007; Majmundar *et al.* 2010; Henke *et al.* 2011; Koeppen *et al.* 2011). For *in vitro* hypoxia studies, the sympathetic ganglion-like clonal rat pheochromocytoma (PC12) cells (Greene and Tischler 1976), which are O_2_-sensitive (Zhu *et al.* 1996; Seta *et al.* 2002), are widely used as a model system. PC12-cells express abundant A2A adenosine receptors (Hide *et al.* 1992; van der Ploeg *et al.* 1996; Arslan *et al.* 1999), which have been shown to affect these cellular responses to hypoxia (Kobayashi *et al.* 1998; Kobayashi and Millhorn 1999). Numerous studies in this cell line, but also in other neuronal cell models, for example, primary cerebellar granule neurons have shown that purine nucleosides have neuroprotective functions in cells, which were subjected to chemical- (Litsky *et al.* 1999; Bocklinger *et al.* 2004; Heftberger *et al.* 2005; Tomaselli *et al.* 2005a; b) and physiological- (Kobayashi and Millhorn 1999; Frizzo *et al.* 2002; Chang *et al.* 2008; zur Nedden *et al.* 2008; Oleskovicz *et al.* 2008; Thomazi *et al.* 2008; Tomaselli *et al.* 2008; Thauerer *et al.* 2010; Dal-Cim *et al.* 2011) hypoxia (Table 1). Results in these models confirmed an important role for A1R as well as A2AR, because purine-mediated rescue was inhibited by A1R (in primary cerebellar granule neurons) or A2AR-antagonists (in neuronal PC12 cells) respectively (Heftberger *et al.* 2005; Tomaselli *et al.* 2005a).

**Table 1:** Key molecules in Purine nucleoside-mediated signal transduction in hypoxic neuronal cells. This table summarizes *in vitro* data, collected from neuronal/hypoxia experiments. Data are separated in the effects of purine nucleosides (adenosine, inosine and guanosine) on (i) viability and (ii) neurite outgrowth

Purine nucleoside	Experimental model	Proposed key molecule	Reference
Viability studies			
Adenosine–NECA	PC12 cells, 1% O_2_	Ca^2+^ homeostasis	Kobayashi and Millhorn (1999)
Adenosine	Cerebellar granule neurons, rotenone		Bocklinger *et al.* (2004)
Adenosine	Cerebellar granule neurons, rotenone	AR (DPCPX), ENT (NBTI)	Heftberger *et al.* (2005)
Adenosine	PC12 cells, rotenone	AR (CSC)	Tomaselli *et al.* (2005a)
Adenosine	PC12 cells, rotenone	ENT (NBTI)	Tomaselli *et al.* (2005a)
Adenosine	PC12 cells, rotenone	PI3K (LY294002)	Tomaselli *et al.* (2005a)
Adenosine	PC12 cells, rotenone	MAPK (PD098059, U0126)	Tomaselli *et al.* (2005b)
Adenosine	PC12 cells, 1% O_2_	MAPK (PD098059, siRNA)	Tomaselli *et al.* (2008)
Adenosine	PC12 cells, 1% O_2_	HIF-1α (siRNA)	zur Nedden *et al.* (2008)
Adenosine	Cerebellar granule neurons, 1% O_2_	MAPK (siRNA)	Tomaselli *et al.* (2008)
Adenosine	Cerebellar granule neurons, 1% O_2_	HIF-1α (siRNA)	zur Nedden *et al.* (2008)
Inosine	Murine spinal cord, rotenone		Litsky *et al.* (1999)
Inosine	Cerebellar granule neurons, rotenone		Bocklinger *et al.* (2004)
Inosine	Cerebellar granule neurons, rotenone	AR (DPCPX), ENT (NBTI)	Heftberger *et al.* (2005)
Inosine	PC12 cells, rotenone	AR (CSC)	Tomaselli *et al.* (2005a)
Inosine	Cerebellar granule neurons, 1% O_2_	MAPK (siRNA)	Tomaselli *et al.* (2008)
Inosine	Cerebellar granule neurons, 1% O_2_	HIF-1α (siRNA)	zur Nedden *et al.* (2008)
Inosine	PC12 cells, 1% O_2_	MAPK (PD098059)	Tomaselli *et al.* (2008)
Inosine	PC12 cells, 1% O_2_	HIF-1α (siRNA)	zur Nedden *et al.* (2008)
Guanosine	Murine spinal cord, rotenone	Purine nucleoside phosphorylase	Litsky *et al.* (1999)
Guanosine	Cortical slices, OGD		Frizzo *et al.* (2002)
Guanosine	Cerebellar granule neurons, rotenone		Bocklinger *et al.* (2004)
Guanosine	Cerebellar granule neurons, rotenone	ENT (NBTI)	Heftberger *et al.* (2005)
Guanosine	PC12 cells, rotenone	AR (CSC)	Tomaselli *et al.* (2005a)
Guanosine	SH-SY5Y cells, OGD		Chang *et al.* (2008)
Guanosine	Hippocampal slices, OGD-reox.		Thomazi *et al.* (2008)
Guanosine	Hippocampal slices, OGD-reox.	PKA, PKC, MEK, PI3K	Oleskovicz *et al.* (2008)
Guanosine	PC12 cells, 1% O_2_	PRK 1(siRNA)	Thauerer *et al.* (2010)
Guanosine	Cerebellar granule neurons, 1% O_2_	PRK 1(siRNA)	Thauerer *et al.* (2010)
Guanosine	Hippocampal slices, OGD-reox.	Ca^2+^-activated K^+^ channels, PI3K, AKT	Dal-Cim *et al.* (2011)
Neurite studies			
Adenosine	Cerebellar granule neurons, rotenone		Bocklinger *et al.* (2004)
Adenosine	Cerebellar granule neurons, rotenone		Heftberger *et al.* (2005)
Adenosine	PC12 cells, 1% O_2_	MAPK (PD098059, siRNA)	Tomaselli *et al.* (2008)
Adenosine	PC12 cells, 1% O_2_	HIF-1α (siRNA)	zur Nedden *et al.* (2008)
Adenosine	PC12 cells, 1% O_2_	AR (SCH-58261)	Thauerer *et al.* (2010) and unpublished data
Inosine	Cerebellar granule neurons, rotenone		Bocklinger *et al.* (2004)
Inosine	Cerebellar granule neurons, rotenone		Heftberger *et al.* (2005)
Inosine	PC12 cells, 1% O_2_	MAPK (PD098059, siRNA)	Tomaselli *et al.* (2008)
Inosine	PC12 cells, 1% O_2_	HIF-1α (siRNA)	zur Nedden *et al.* (2008)
Inosine	Dorsal root ganglion neurons	Mstb3, MAPK	Lorber *et al.* (2009)
Inosine	PC12 cells, 1% O_2_	AR (SCH-58261)	Thauerer *et al.* (2010) and unpublished data
Guanosine	Cerebellar granule neurons, rotenone		Bocklinger *et al.* (2004)
Guanosine	Cerebellar granule neurons, rotenone		Heftberger *et al.* (2005)
Guanosine	PC12 cells, 1% O_2_	PRK 1 (siRNA)	Thauerer *et al.* (2010)
Guanosine	Cerebellar granule neurons, 1% O_2_	PRK 1 (siRNA)	Thauerer *et al.* (2010)
Guanosine	PC12 cells, 1% O_2_	AR (SCH-58261)	Thauerer *et al.* (2010) and unpublished data

In hypoxic PC12 cells, viability is predominantly rescued by adenosine, whereas guanosine is more supportive of neurite outgrowth (Kobayashi *et al.* 1998; Tomaselli *et al.* 2005a). Hypoxia-induced membrane responses of PC12 cells are likely to be mediated via activation of the A2A adenosine receptors, and elevation of cAMP and inhibition of the A2A receptor itself induced death of PC12 cells (Kobayashi *et al.* 1998; Arslan *et al.* 1999; Tomaselli *et al.* 2005a). Therefore, it is not surprising that PKA is required for the A_2_ receptor modulation of both voltage-sensitive potassium *I*_K(V)_ and calcium *I*_Ca_ currents in PC12 cells (Kobayashi *et al.* 1998) and that pharmacological inhibition of protein kinase A (PKA) with H89 superinduced chemical hypoxia-mediated cell death and inhibited the rescue of hypoxic PC12 cells by purine nucleosides (Tomaselli *et al.* 2005a). The role of nucleoside transport varies for different purine nucleosides and cell types. In PC12 cells, the inhibition of nucleoside transport with *S*-(4-nitrobenzyl)-6-thioinosine caused an increase in adenosine-mediated rescue of viability (Tomaselli *et al.* 2005a), presumably due to increased A2A receptor-mediated signaling (Parkinson *et al.* 2000). Our own results therefore confirm the hypothesis that adenosine mainly acts via adenosine receptor-mediated signaling mechanisms, whereas many aspects of the mechanisms involved in inosine- and guanosine-based protection still remain unclear. However, A1R-expressing primary cerebellar granule neurons were more effectively rescued by the adenosine metabolite inosine, than adenosine and guanosine (Bocklinger *et al.* 2004; Heftberger *et al.* 2005), whereby adenosine- and inosine-mediated rescue was sensitive to an A1R antagonist (8-cyclopentyl-1, 3-dipropylxanthine) whereas guanosine was largely unaffected (Heftberger *et al.* 2005). Nucleoside transport is apparently important for adenosine-, inosine- and guanosine-mediated rescue of hypoxic A1R positive cerebellar granule neurons (Heftberger *et al.* 2005).

**Figure 2:**
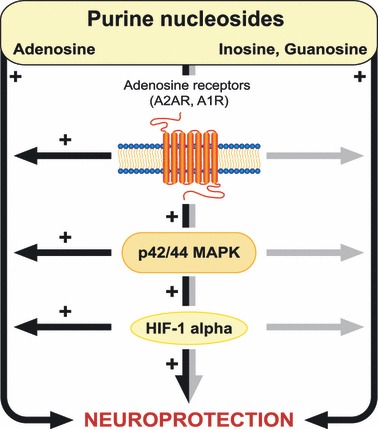
Key-signaling modules in purine-mediated protection of hypoxic neurons.In brain mainly A1 and A2A G-protein coupled adenosine receptors are expressed (Fredholm *et al.* 2005a; b; Wei *et al.* 2010). Two models were established, PC12 cells (predominantly A2AR-positive) and cerebellar granule neurons (A1R-positive), in which the significance of the purine nucleosides adenosine (left side) and inosine and guanosine (right side) in protection of hypoxic neurons was proved (Bocklinger *et al.* 2004; Heftberger *et al.* 2005; Tomaselli *et al.* 2005a, b, 2008; zur Nedden *et al.* 2008; Thauerer *et al.* 2010). The importance of A1R and A2AR was confirmed using specific receptor antagonists (Heftberger *et al.* 2005; Tomaselli *et al.* 2005a). Purine nucleoside-mediated neuroprotection also critically involves the activation of mitogen-activated protein kinases (MAPKs; Tomaselli *et al.* 2008), hypoxia-inducible factor-1 (HIF-1α) (zur Nedden *et al.* 2008), and their interconnection (B. Thauerer, unpublished data). The adenosine receptor MAPK-HIF-1α module plays therefore a dominant role in adenosine-mediated protection (black bars), and takes also part in inosine- and guanosine-mediated neuroprotection of hypoxic neuronal cells (grey bars).

The p42/44 mitogen-activated protein kinase (MAPK) pathway, serine–threonine kinases constitute a convergent pathway for the regulation of multiple modalities involved in O_2_ sensing (Seta *et al.* 2002). They are part of a signaling module that transduces signals from the cell membrane to the nucleus in response to a vast range of external stimuli (Irving and Bamford 2002; Colucci-D'Amato *et al.* 2003; Cheung and Slack 2004; Wada and Penninger 2004) and regulate proliferation, neuronal survival, differentiation, long-term memory and synaptic plasticity and apoptosis (Boulton *et al.* 1991; Segal and Greenberg 1996; Alessandrini *et al.* 1999; Johnson and Lapadat 2002; Sweatt 2004; Wada and Penninger 2004). Emerging evidence suggests that MAPKs are highly related to processes that promote neuron survival (e.g. by neuroprotective growth factors (Nicole *et al.* 2001), and plasticity (Impey *et al.* 1999). Stimulation of the adenosine receptors A1R, A2A- and A2BR was shown to activate MAPK (Sexl *et al.* 1997; Dickenson *et al.* 1998; Arslan and Fredholm 2000; Fredholm *et al.* 2001a; Charles *et al.* 2003; Schulte and Fredholm 2003a; b; Tomaselli *et al.* 2008). Authors reflect on different activation pathways of MAPK, as diverse as coupling of ARs to G_12/13_ proteins instead of *G*_s_ or *G*_s_- and cAMP-independent affects on MAPK involving the Ras module (Sexl *et al.* 1997; Seidel *et al.* 1999; Schulte and Fredholm 2003b). Along this line another group (Faure *et al.* 1994) showed transiently expressed A1R in COS-7 cells mediated MAPK activation via release of βγ subunits. Although it appears from these and from many other cases (Hetman and Gozdz 2004), that MAPK activation is neuroprotective, mediating the effects of several extrinsic survival signals, MAPK activation in hypoxia/ischemia still remains a controversial issue. MAPKs are activated by small increases in calcium during survivable degrees of hypoxia (Minet *et al.* 2000; Bickler and Donohoe 2002) and studies in perinatal cerebral hypoxia–ischemia showed MAPK activation in neurons, mainly in cells displaying signs of damage (Wang *et al.* 2003). Authors therefore debate, whether MAPK is either trying, unsuccessfully, to rescue cells, or actually contributing to harmful cell signals (Wang *et al.* 2003). Recent results, however, suggested a vital role of the MAPK pathway in purine nucleoside-mediated protection of neuronal cells and primary neurons following hypoxic insult (Tomaselli *et al.* 2005b, 2008). In cells subjected to hypoxia, an increased phosphorylation of MAPK, was detected, that was further increased upon addition of purine nucleosides. Vice versa, upon blocking this pathway with a pharmacological inhibitor of MEK-1 (PD098059) viability and neurite outgrowth were decreased (Tomaselli *et al.* 2008). Further evidence came from experiments with small interference RNA constructs. Knockdown of MAPK severely affected purine nucleoside-mediated rescue of hypoxic PC12 cells and cerebellar granule neurons (Tomaselli *et al.* 2008; Fig. 2).

MAPK was shown to positively modulate the hypoxia-inducible factor-1 alpha (HIF-1α) by phosphorylation control (Minet *et al.* 2000). HIF-1α, a transcription factor that plays an essential role in cellular and systemic responses to hypoxia, appears especially interesting and relevant for hypoxia induced signaling. HIF-1α is a heterodimer composed of a 120-kDa HIF-1α subunit complexed to a 91- to 94-kDa HIF-1-β subunit. Under hypoxic conditions HIF-1α is stabilized and constitutes a key role in the cellular defense against hypoxic injury, including the regulation of genes involved in energy metabolism, angiogenesis, and apoptosis (Sitkovsky and Lukashev 2005; Semenza 2011). Direct HIF-1 target genes are involved in energy metabolism and cell viability and thus HIF-1 is causally involved in human disease pathophysiology such as cerebral ischemia (Semenza 2000). Adenosine was hypothesized to have the ability to engage HIF-1 activation towards the cellular and systemic responses to hypoxia it mediates (Sitkovsky 2009). Fitting to these data, it was later reported that the nuclear HIF-1α signal in neuronal cells is increased by adenosine and that HIF-1α is apparently critical for purine-mediated neuroprotection (zur Nedden *et al.* 2008). Authors conclude from their results that the adenosine receptor/MAPK/HIF-1α pathway is tightly interwoven as proven by pharmacological inhibition or siRNA knockdown and plays a critical role for adenosine- and to a lesser degree also for inosine and guanosine-mediated neuro-protection (Fig. 2).

Next to the clear-cut effects of adenosine receptor-mediated activation of MAPK-HIF-1α, neuroprotection of hypoxic neuronal cells apparently does involve other pathways that deserve future attention. Amongst purine nucleosides guanosine attracted attention for its strong neurite-stimulating capacity (Bau *et al.* 2005; Jiang *et al.* 2007; Schmidt *et al.* 2007; Chang *et al.* 2008; Rathbone *et al.* 2008; Thauerer *et al.* 2010). Neuroprotective effects of guanosine were reported to involve an augmentation of glutamate uptake modulated by K^+^ channels and the activation of the phosphatidylinositol 3-kinase/Akt pathway (Dal-Cim *et al.* 2011). Recently, another protein kinase, namely protein kinase N alpha (PKNα)/protein kinase C-related kinase1 (PRK1) (Mellor and Parker 1998; Mukai 2003), made a name of itself in purine-mediated neuroprotection (Tomaselli *et al.* 2005a; Thauerer *et al.* 2010). PRK1 is a lipid-activated serine/threonine protein kinase and a member of the protein kinase C superfamily (Mellor and Parker 1998; Mukai 2003) of potential key regulators orchestrating physiological responses, and is involved in regulation of the actin cytoskeleton (Modha *et al.* 2008). PRK1 is activated by interacting with the Rho and Rac families of small G-proteins and arachidonic acid, or by caspase cleavage (Takahashi *et al.* 1998; Lu and Settleman 1999; Mukai 2003). Adenosine, inosine and guanosine up-regulated its activity in hypoxic neuronal cells (Tomaselli *et al.* 2005a; Thauerer *et al.* 2010). Vice versa, loss of functional PRK1 initiated a significant loss of viability and inhibition of neurite formation (Thauerer *et al.* 2010), which apparently involved a disturbance of the F-actin-associated cytoskeleton and the expression of the plasticity protein growth-associated protein-43 (Thauerer *et al.* 2010). An up-regulation of growth-associated protein-43 was also reported for inosine (Petrausch *et al.* 2000). To what extent inosine's ability to induce neurite outgrowth (Zurn and Do 1988; Benowitz *et al.* 1998) is due to the activity of Mst3b, a Ste-20-like purine-sensitive protein kinase (Irwin *et al.* 2006; Lorber *et al.* 2009) or on PRK1 remains to be shown (Table 1).

## Conclusion

Hypoxic–ischemic brain injury starts with the insult but extends into a recovery–reperfusion period (Barone and Feuerstein 1999; Lipton 1999; White *et al.* 2000; Hertz 2008; Macrez *et al.* 2011). In case of prolonged ischemia, restricted blood flow leads to a reduction in ATP, causing severe impairment of cellular function by disruption of ATP-dependent processes. Brain exposure to hypoxia in ischemia/reperfusion injuries often causes devastating and irreversible loss of function (Chen *et al.* 2002) and is linked to long term neurological shortages (Berger and Garnier 1999; El-Khodor and Boksa 2000). In parallel hypoxia leads to the decreased production and enhanced breakdown of purine nucleotides to purine nucleosides (Jurkowitz *et al.* 1998; Sitkovsky *et al.* 2004; Fredholm *et al.* 2007; Fredholm 2010). Earlier studies showed that in response to hypoxia, adenosine is produced intracellularly and released into the medium (Meghji *et al.* 1989; Lloyd *et al.* 1993; Parkinson and Xiong 2004; Takahashi *et al.* 2010), from where it triggers different actions through the activation of ARs (Fredholm *et al.* 1994, 2001a; Schulte and Fredholm 2003b). Growing evidence suggests that purine nucleotides and nucleosides might act as trophic factors in both the central and peripheral nervous systems and are involved in the regulation of the nervous system's development and plasticity (Neary *et al.* 1996). Adenosine was reported to act as a powerful endogenous neuroprotectant during ischemia-induced energy failure by decreasing neuronal metabolism and increasing cerebral blood flow, and by playing a variety of different roles as an intra- and intercellular messenger (Dunwiddie and Masino 2001; Fredholm *et al.* 2005a; b). Guanosine and inosine the like, were shown to induce neurite outgrowth (Benowitz *et al.* 1998; Rathbone *et al.* 2008) and *in vivo* studies demonstrated inosine's ability to stimulate neurons to extend new projections to denervated areas in adult rats with unilateral cortical infarcts (Chen *et al.* 2002). *In vitro* studies confirmed the amazing neuroprotective capability of purine nucleosides in several neuronal hypoxia systems e.g PC12 cells and cerebellar granule neurons (Bocklinger *et al.* 2004; Heftberger *et al.* 2005; Tomaselli *et al.* 2005a, b, 2008; zur Nedden *et al.* 2008; Thauerer *et al.* 2010) and prompted the investigation of purine-mediated hypoxia sensitive signaling. Amongst the multiple pathways and sophisticated mechanisms that have evolved and regulate gene expression during hypoxia, the MAPK module constitutes a convergent pathway for the regulation of multiple modalities involved in O_2_ sensing (Seta *et al.* 2002). Stimulation of the A1R, A2A- and A2BR was shown to activate MAPK (Sexl *et al.* 1997; Dickenson *et al.* 1998; Arslan and Fredholm 2000; Fredholm *et al.* 2001a; Charles *et al.* 2003; Schulte and Fredholm 2003a; b; Tomaselli *et al.* 2008) and recent results suggested a vital role of the MAPK pathway plays in purine nucleoside-mediated protection of neuronal cells following hypoxic insult (Tomaselli *et al.* 2005b, 2008). These results are very relevant to understand the mechanisms by which purine nucleosides modulate neuronal signaling and should support the therapeutic approaches investigated by other groups, which claim that brief ischemia activates MAPK whereas its blockade inhibits ischemic tolerance (Meller *et al.* 2005). MAPK activation may thus act as a defensive mechanism that helps to compensate for deleterious effects of a damaging insult (Hetman and Gozdz 2004). Amongst MAPK-associated (Minet *et al.* 2000) downstream effector molecules the transcription factor HIF-1α appears to be most interesting. Adenosine was hypothesized to collaborate with HIF-1α in triggering the production of immunosuppressive molecules (Sitkovsky 2009). Likewise adenosine augmented hypoxia-mediated HIF-1α translocation to the nucleus and HIF-1α was shown to be critical for purine-mediated neuroprotection (zur Nedden *et al.* 2008).

Many stroke patients fail clinical time windows for acute effective treatment, hence making approaches that promote repair and recovery essential for integrated stroke therapy (Moskowitz *et al.* 2010). Growing evidence suggests that the biological processes underlying stroke are driven by the interaction of neurons, glia, vascular cells, and matrix components; all actively participating in tissue injury and repair and therefore trophic factor treatments that amplify and augment endogenous processes of neuroplasticity are pre-destined to support recovery (Moskowitz *et al.* 2010). Furthermore, the detection of continuous neurogenesis in the adult mammalian brain has encouraged a new perception of the plasticity of the mature nervous system (Ming and Song 2011). Thus, as data on the competence of purine nucleosides to support neuroprotection and regeneration accumulate, increasing levels of pro survival proteins may be a promising new strategy to reduce cell damage after ischemia (Cao *et al.* 2002). In light of recent developments for adenosine in epilepsy (Van Dycke *et al.* 2011), purine nucleoside augmentation techniques or localized delivery may facilitate possible approaches for neuroprotection and/or enhanced neuroregeneration in stroke.

## Abbreviations

AR: adenosine receptor 1, 2A, 2B, 3
ENT: equilibrative nucleoside transporter
*G*_i_: inhibiting G-protein
*G*_s_: stimulating G-protein
HIF-1α: hypoxia-inducible factor-1 alpha
MAPK: p42/44 mitogen-activated protein kinase, also ERK1/2
PI3-K: phosphatidylinositol 3-kinase
PKA: protein kinase A
